# Metal-Free Carbon-Based Materials: Promising Electrocatalysts for Oxygen Reduction Reaction in Microbial Fuel Cells

**DOI:** 10.3390/ijms18010025

**Published:** 2016-12-24

**Authors:** Sandesh Y. Sawant, Thi Hiep Han, Moo Hwan Cho

**Affiliations:** School of Chemical Engineering, Yeungnam University, Gyeongsan, Gyeongbuk 712-749, Korea; sandeshcsmcri@gmail.com (S.Y.S.); hanhiepnhim@gmail.com (T.H.H.)

**Keywords:** bioelectrochemical systems, electrocatalysis, bio-energy, waste treatment, carbon-based cathodes

## Abstract

Microbial fuel cells (MFCs) are a promising green approach for wastewater treatment with the simultaneous advantage of energy production. Among the various limiting factors, the cathodic limitation, with respect to performance and cost, is one of the main obstacles to the practical applications of MFCs. Despite the high performance of platinum and other metal-based cathodes, their practical use is limited by their high cost, low stability, and environmental toxicity. Oxygen is the most favorable electron acceptor in the case of MFCs, which reduces to water through a complicated oxygen reduction reaction (ORR). Carbon-based ORR catalysts possessing high surface area and good electrical conductivity improve the ORR kinetics by lowering the cathodic overpotential. Recently, a range of carbon-based materials have attracted attention for their exceptional ORR catalytic activity and high stability. Doping the carbon texture with a heteroatom improved their ORR activity remarkably through the favorable adsorption of oxygen and weaker molecular bonding. This review provides better insight into ORR catalysis for MFCs and the properties, performance, and applicability of various metal-free carbon-based electrocatalysts in MFCs to find the most appropriate cathodic catalyst for the practical applications. The approaches for improvement, key challenges, and future opportunities in this field are also explored.

## 1. Introduction

Over the past decade, fundamental and applied research on microbial fuel cells (MFCs) has become a “hot topic” with the increasing concern for alternative energy sources, waste management, and global climate change. MFCs are a green technology that can directly convert the organic energy of wastewater to electricity [1]. Similar to chemical fuel cells, MFCs are composed of an anode and a cathode, which are often separated by a proton-exchange membrane (PEM). In the anode, electrochemically active microorganisms degrade organic matter and respire extracellularly by transferring electrons to the anode [2]. The electrons flow spontaneously through an external circuit to the cathode for reduction reactions. In the cathode, an appropriate electron acceptor is reduced by a reaction with protons and electrons coming from the anode. Microbial fuel cells have many advantages over energy generation from biomass, such as high energy conversion efficiency, room temperature operation, no requirement for gas treatment, and low energy input [3]. Compared to conventional activated sludge processes, MFCs can theoretically achieve a positive energy balance and produce significantly less waste sludge, which further enhances the energy efficiency [4]. Moreover, the electricity produced by MFCs is cleaner than methane in terms of greenhouse effects [5].

On the other hand, to be applied in practice, MFCs need to overcome many limitations, including low power generation and the high cost of materials, such as electrode, membrane, and catalysts in the cathode [6]. The performance of the cathode is considered the main limitation in MFCs. One of the critical challenges is the development of cheap, efficient, and stable cathodic catalyst for the oxygen reduction reaction (ORR) in MFCs. In the MFC cathode, oxygen has been used widely as an electron acceptor because of its high standard redox potential, unlimited availability, and sustainability [7]. In the absence of a catalyst, the kinetic oxygen consumption rate is very slow, which accounts for the large proportion of potential loss [8]. Platinum with high ORR catalytic activity in nature is used widely as a cathode catalyst in MFCs to improve their overall performance. In spite of this fact, the practical applications of Pt-based ORR electrode catalysts are limited by their poisoning sensitivity and the high cost of Pt [9,10]. Therefore, the development of an efficient and low cost catalyst for the ORR has attracted considerable interest in recent years. A huge number of catalysts are investigated, which can be categorized into biocatalysts, carbon-based, metal-based, and electro-conductive polymer-based catalysts [11]. Although many metal-based catalysts were claimed to produce a power density similar to or better than that of Pt, they still suffer from leaching problems [12,13], and result in a low stability [14]. Another major concern of metal-based catalysts is the cost, which is still high and not competitive with carbon-based catalysts [5]. Biocatalysts also have stability issues that depend on the microbial community dynamics and environmental fluctuations. The application of electro-conductive polymer-based catalysts in MFCs was restricted owing to their poor performance compared to Pt/C. Hence, electro-conductive polymers are usually combined with other electrocatalytically active materials to enhance performance [15]. Carbon-based materials—with the high catalytic activity, cost effectiveness, and durability—can fulfill the requirements to be the alternatives to Pt/C and become the most prospective cathodic catalyst for MFC practical applications [16].

A cathode electrode usually consists of the catalyst layer and the supporting material, which generally acts as the diffusion layer, as well as the current collector. Carbon-based materials in different morphologies and shapes have been widely used as catalysts and catalyst supports in cathode electrode of MFCs [17]. Activated carbons (ACs) has higher ORR catalytic activity than carbon black (CB) and have been successful in obtaining power densities similar or higher than to those achieved with commonly used Pt/C cathodes [18]. Activated carbon can be synthesized from different renewable waste materials such as coconut shells, wood chips, and sawdust. Thus, AC is considered to be a more promising and inexpensive alternative cathode material compared to metal-based ORR catalysts. Carbon nanostructures, especially graphene, carbon nanotubes (CNTs) and carbon nanofibers (CNFs), with unique electrical and mechanical properties received great interests in MFC research [11]. Heteroatom-doped carbon nanomaterials are new class of catalysts which can replace Pt/C with efficient ORR catalytic activity in the fuel cells [19]. Heteroatom-doped carbon nanomaterials also well performed multifunctional catalyst and showed the better catalytic activity towards oxygen evolution reaction and hydrogen evolution reaction [19]. Recently, the heteroatom-doped carbon nanomaterials are gaining tremendous interest in the field of ORR catalysts due to their comparable ORR activity with Pt/C and much higher stability after longer use and towards catalyst poising [9,20,21].

Many reviews are available on ORR catalysts in fuel cell and batteries [19,22,23] and in MFCs [5,11,16,24,25] but to the best of the authors’ knowledge, there is no in-depth overview of carbon-based catalysts in MFCs. This paper reviews the recent advances in the development of carbon-based metal-free catalysts in MFCs. In particular, this review provides knowledge of the carbon-based electro-catalysts characteristics with a focus on AC, heteroatom-doped carbon materials, and their superior applications in MFCs to identify the most appropriate cathodic catalyst for practical applications of MFCs. The approaches for improvement, key challenges, and future opportunities in this field will be also explored.

## 2. Mechanism of Oxygen Reduction Reaction in Cathode

The mechanisms of the ORR involve a series of electron transfer process that depend strongly on the type of catalyst used. Generally, the electrochemical reduction of oxygen in an aqueous electrolyte can proceed via two overall pathways (i.e., four-electron pathway Equation (1) and two-electron Equations (2)–(4)). The four-electron pathway, in which oxygen is reduced directly to water, is more favorable than the two-electron pathway, which includes the production of hydrogen peroxide. The four-electron pathway appears to be predominant in noble metal electrocatalysts [25]. In most carbon materials, depending on the catalyst and the preparation method, ORR can follow a two-electron pathway (e.g., CNTs) [26], or a combination of two- and four-electron pathways (such as S-doped graphene [27] and N-doped carbon [28]), or four-electron pathway (Co and N-doped carbon [29]). pH has a strong effect on the catalytic pathway. 

ORR pathway in acidic medium:

Four-electron pathway:

(1) $$O_{2}+4H^{+}+4e^{-}\to 2H_{2}O\;E=1.229V$$

Two-electron pathway:

(2) $$O_{2}+2H^{+}+2e^{-}\to H_{2}O_{2}\;E=0.695V$$

(3) $$H_{2}O_{2}+2H^{+}+2e^{-}\to 2H_{2}O\;E=1.770V$$

(4) $$2H_{2}O_{2}\to 2H_{2}O+O_{2}$$

Experimental and modeling studies suggest that the ORR in a neutral or alkaline catholyte proceeds dominantly via the OH^−^ producing pathway Equations (5)–(8). The OH^−^ accumulated at the catalytic sites can lead to a considerable decrease in the thermodynamic and kinetic performance [30]. Therefore, the effective removal of OH^−^ is a big concern in ORR. The functional groups on the catalyst surface may assist in the OH^−^ transportation and ORR kinetics.

ORR pathway in neutral or basic medium:

Four-electron pathway:

(5) $$O_{2}+2H_{2}O+4e^{-}\to 2{\mathrm{OH}}^{-}\;E=0.401V$$

Two-electron pathway:

(6) $$O_{2}+H_{2}O+2e^{-}\to {\;\mathrm{HO}}_{2}^{-}+{\;\mathrm{OH}}^{-}\;E=-0.065V$$

(7) $${\mathrm{HO}}_{2}^{-}+H_{2}O+2e^{-}\to 3{\mathrm{OH}}^{-}\;E=0.867V$$

(8) $$2{\mathrm{HO}}_{2}^{-}\to 2{\mathrm{OH}}^{-}+O_{2}$$

The rotating disk electrode (RDE) is the most widely used technique for screening the ORR activity [31]. The evaluation of a material is based on the kinetic rates and reaction pathways under non-mass transfer limited conditions. Catalyst powders are typically dispersed in a water/alcohol mixture forming a uniform ink, which is then deposited on glassy carbon (GC) electrodes to form the catalyst films. To mitigate the mass transfer effect during the ORR activity measurements, GC electrodes are rotated to increase the mass transfer rates of O_2_ at the electrode surface. The intrinsic activity (kinetic current without mass transfer effect) of the catalysts can be derived using the Koutecky–Levich equation (Equation (9)) [32].

(9) $$\frac{1}{j}=\frac{1}{j_{k}}+\frac{1}{j_{d}}$$

(10) $$j_{d}=0.62nFC_{O_{2}}D_{O_{2}}^{2/3}v^{-1/6}w^{1/2}$$

where *j* is the measured limiting current density, *j_k_* is the kinetic current density, *j_d_* is the diffusion-limited current density, *F* is the Faraday constant (*F* = 96,485 C/mol) [9], *C_O_*_2_ (1.2 × 10^−6^ mol/cm^3^) is the saturated concentration of oxygen in 0.1 M KOH [33,34], *D_O_*_2_ (1.9 × 10^−5^ cm^2^/s) is the diffusion coefficient of oxygen in 0.1 M KOH [35,36], ν is the kinematic viscosity of 0.1 M KOH (*ν* = 0.01 cm^2^/s) [36], and ω is the electrode rotation rate in rad/s. The number of electrons transferred is calculated from the slope of the Koutecky–Levich equation and can provide the information on the ORR pathway. For example, on commercial Pt/C, the number of electrons transferred is approximately 3.96 [28], which suggests that oxygen is reduced via a four-electron transfer pathway. RDE analysis was used to study the ORR catalysis of many materials, where the RDE results were well correlated with the MFC performance [37,38,39]. Note that RDE measurement grants optimal conditions without the usual diffusion limitations found in MFC cathodes. Therefore, some studies reported an inverse correlation between the RDE and MFC [27,28,40]. Further information from CV may be helpful in these cases.

## 3. Cathode Control Energy Loss in Microbial Fuel Cells

There are three major losses that occur in the cathode, which control the MFCs performance, including activation losses, ohmic losses, and mass transport losses. Current production in MFCs depends largely on the kinetics of the reduction that takes places at the cathode [41], which is limited by the activation energy barrier. These cathodic activation losses are especially apparent at low current densities. The oxygen reduction is the most common cathodic reduction due to its high standard potential and unlimited availability [16]. On the other hand, the limited solubility and slow kinetic rate of oxygen reduction due to the high activation energy (498 kJ/mol) cause the high cathodic overpotential [8]. This appears to cause the dominant limitation in MFCs.

Several approaches to reduce the activation losses in the cathode are the use of mediators, electrode modification with catalysts, and optimizing the operational conditions within the cathodic compartment [41]. The purpose of these approaches is to enhance the kinetics of the ORR. The use of mediators, such as ferricyanide, permanganate, dichromate, and persulfate, can increase the reduction rate on the electrode surface but they are potentially toxic, and impractical for long-term operation [42]. The most perspective approach is the use of a ORR catalyst to improve the reduction kinetic performance. Catalysts can decrease the energy barrier or increase the ORR interface area [41], thereby enhancing the performance of MFCs.

## 4. Requirements of an Ideal Oxygen Reduction Reaction Catalyst

An ideal ORR catalyst used in MFCs is expected to show high catalytic activity, be cost-effective, and have high durability.

### 4.1. High Catalytic Activity

The most vital requirement of a material to be used as an ORR catalyst is that it needs to possess high catalytic activity. Overall, the activity is related directly to the electrical conductivity. The improvement in electrical conductivity facilitates electron transfer and enhances power generation in MFCs [43]. In addition, the activity of the catalysts towards the ORR depends on the nature and morphology of the materials. In the case of metal-based catalysts, their ORR activity relies on their d-band center vacancies [44]. The ORR catalytic activity of polymer materials is caused by their specific electronic structure, in which oxygen is chemisorbed reversibly through a bridge adsorption model at some carbon centers [45]. The high ORR catalytic activity of carbon-based materials was attributed to their morphology and characteristics, such as electrical conductivity, specific surface area, and cell structure. The high surface area provides additional active sites for the ORR, and facilitates the transfer of electrolyte ions and O_2_ during the ORR [46]. The increase in surface area of CNFs from 275 to 2100 m^2^/g by an alkaline treatment consequently enhanced the MFC performance by 79% [47]. The pore structure of the catalyst layer also plays a crucial role in determining the ORR activity. Macro- and mesopores of the AC are beneficial for oxygen transfer while micropores might provide additional active sites for the ORR [48].

### 4.2. Cost Effectiveness

Currently, the cathode material costs account for 47%–75% of the overall capital costs of MFC [10]. The ideal ORR catalysts used in MFCs are expected to be cost-effective because MFCs are engineered primarily for wastewater treatment. Therefore, the capital and maintenance costs should be comparable to conventional treatment technologies. At this stage of development, the energy generated by MFCs is used mainly to balance the energy consumption rather than to generate additional economic benefits, further highlighting the importance of the economic feasibility of cathode catalysts. Hence, the properties associated with practicality, including simple and large-scale synthesis, low cost, and high durability should be given priority when developing ORR catalysts for MFC applications.

### 4.3. Long-Term Stability

In real MFC applications, some aqueous contaminants or reaction intermediates, such as chloride and sulfide may lead to catalyst deactivation during long-term operation, due to dissolution, oxidation, poisoning, or fouling. In particular, the accumulation of OH^−^ at the vicinity of the ORR cathode usually leads to a considerable drop in the thermodynamic and kinetic performance [30]. Therefore, catalysts should possess not only a good catalytic activity, but also high selectivity and durability [24].

## 5. Limitations of Metal-Based Catalysts

Platinum has been used widely as the most common precious metal catalyst in cathode materials of MFCs because it has a favorably low overpotential for oxygen reduction. Logan et al. reported one order of magnitude increases in the power output of double chamber MFCs with a Pt-coated cathode compared to the plain carbon electrode [49]. Although Pt has excellent catalytic ability, its relatively high cost limits its application. Efforts have been devoted to address this problem by reducing the amount of Pt necessary to coat the cathode electrode [50] or replacing Pt with non-precious metal catalysts. Compared to Pt, performance of non-precious metals in cathodes are comparable (Co- and Fe-based materials) or even much higher, approximately four times improvement (PbO_2_ [12]). More importantly, the cost per unit power can be reduced by one half. On the other hand, the possibility of lead leaching from the cathode is a potential hazard that limits the use of PbO_2_ as a cathode catalyst in MFCs. Such a limitation can be overcome by improving the coating techniques and binding materials, which would enhance the stability of the catalyst.

However, metal-based catalysts are generally susceptible to adverse environmental conditions that can occur in MFCs as a result of chemical reactions, biological activity, and changes in the catholyte composition. For example, their catalytic activity is reduced in sulfide- or chloride-rich environments. Schmidt et al. used a thin-film rotating disk Pt-electrode in a liquid electrolyte to demonstrate that adsorbed Cl^−^ ions act as site blocking species [51]. This effect reduced the active area available for oxygen reduction and altered the reaction pathway toward the production of H_2_O_2_.

Moreover, metal-based catalysts are also sensitive to high cathodic pH values, which is a common phenomenon that occurs in MFCs due to the crossover of cations through the membrane to the cathode compartment. Zhao et al. studied the influence of the catholyte composition on the performance of Fe- and Co-based cathode catalysts for oxygen reduction [52]. They demonstrated that lowering the concentration of a phosphate buffer catholyte (pH 3.3) from 500 to 50 mM reduced the performance of a pyr-FePc-modified electrode by 40%. With all of the above limitations, metal-based catalysts seem not to be ideal ORR catalysts for large scale application of MFCs.

## 6. Why Carbon-Based Catalysts?

### 6.1. High Catalytic Activity and Durability

Carbon materials meet all the requirements to be an ideal ORR catalyst. They offer high conductivity, high durability, high mechanical strength, and reasonable cost. Thus, they are becoming the most promising alternatives to metal-based catalysts and are the most common materials used for cathode electrodes in MFCs [16]. Carbon materials also benefit from the large specific surface area, which provides additional active sites for the ORR [53]. Their superior performance as ORR catalysts in MFCs is also due to their low internal resistance [54] or a combination of large surface area, open structure, and better conductivity [55]. Therefore, most carbon-based metal-free ORR catalysts show similar or better ORR activities to Pt/C in MFCs [5,27]. In addition to the high catalytic ORR activity, carbon-based catalysts, such as N-doped carbon materials, show exceptional cycling durability and resistance to catalytic poisoning with methanol, CO, and sulfide [56]. Similarly, CNFs can tolerate sulfide poisoning and biofilm growth under long-term operation, whereas the commercial Pt/C cathode can be deteriorated severely [57]. Activated carbon as an ORR catalyst exhibits excellent electrochemical durability. In the chronoamperometry test, a N-doped AC cathode showed only a 30% decrease in current density after 7 h compared to 73% for the Pt/C cathode [58].

### 6.2. Cost Effectiveness

One of the advantages of using carbon-based catalysts over metal-based catalysts is the cost effectiveness. In terms of cost, carbon materials are far superior to metal-based catalyst. Interestingly, carbon-based catalysts can be prepared from a variety of sustainable precursors, including natural or waste materials [59,60,61,62]. Therefore, the utilization of sustainable carbon-based catalysts as ORR catalysts is an outstanding approach to reduce both the capital costs of MFCs as waste discharge to environment. A previous study reviewed in detail the cost of some carbon-based catalysts used in MFCs [5]. Overall, all the carbon-based catalysts reviewed obtained higher cost effectiveness than commercial Pt/C (1 mW/$), highlighting their feasibility for practical MFC applications. According to the estimated price, loading rate, and power production, the cost normalized maximum power density N/P co-doped cellulose carbon could reach up to 143 mW/$ [63], polyaniline (PANI)/multi-walled CNTs (MWCNTs) composite reached 31 mW/$ [64]. The lower cost effectiveness group included graphite at 12 mW/$ [65], and graphene at 10 mW/$ [66]. CNFs/CNTs accounted for the lowest cost effectiveness among ORR carbon-based catalysts but still 2.6 times more effective than Pt/C [47]. Noticeably, the maximum power density normalized by the material cost of AC is highest with 1210 mW/$, which is 1210 times higher than that of commercial Pt/C. Activated carbon also exhibits very high stability with only a 15% decrease after 16 months operation in MFCs [67]. Therefore, AC appears to be the most promising catalyst for practical applications of MFCs.

## 7. Carbon-Based Oxygen Reduction Reaction Catalysts

### 7.1. Carbon Black

**Figure 1:**
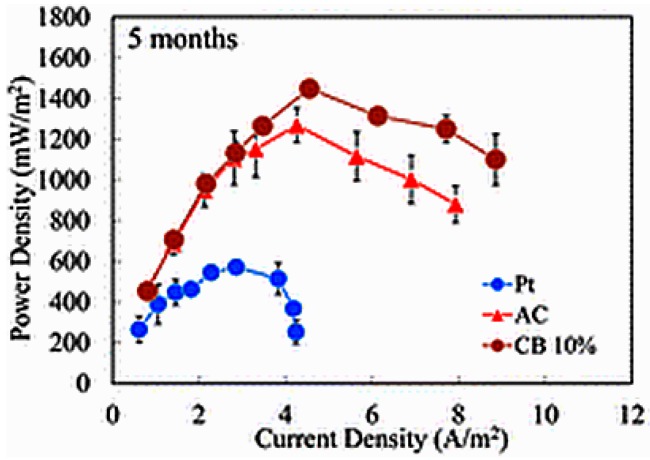
Power density and current density curve for microbial fuel cells (MFCs) operated for 5 months using Pt, activated carbon (AC), and 10% carbon black (CB)-blended AC (Reprinted with permission from Ref. [43]. Copyright (2014) American Chemical Society).

Carbon black is produced commercially from heavy petroleum products possessing good electrical conductivity and high surface area (lower than AC) and is used mostly as a reinforcing material. In the case of power production, CB is used exclusively as a catalyst support in low-temperature fuel cells. Carbon black possesses poor ORR catalytic activity compared to Pt or other metal-based catalysts [68]. In contrast, it is applied mostly as a comparative ORR catalyst or catalyst support in MFCs owing to its high electrical conductivity and moderate surface area [69]. Various grades of CB are available in the market depending on their physicochemical properties. Mshoperi et al. carried out a voltammetry study of different kinds of commercial CB samples and observed a decrease in the ORR overpotential up to 270 mV compared to the GC electrode, which could be reduced further to 600 mV if used in combination with FePc [70]. According to their observations, the decrease in the ORR potential with CB was independent of its surface area and particle size. A four-fold increase in maximum power density (4.07 to 17.32 mW/m^2^) could be achieved using FePc with CB compared to carbon paper (CP). The addition of CB with AC is an efficient way to enhance the ORR activity and the overall performance of the MFC through improvements in conductivity. The incorporation of an appropriate amount of CB achieved an almost five-fold enhancement in the electrical conductivity of a polymer composite [71]. The 10% addition of CB with AC improved the power density of MFC by 16% (1560 ± 40 mW/m^2^) compared to those with the plain AC cathode (1340 ± 120 mW/m^2^) and 7% higher than the MFCs with a Pt cathode (1460 ± 10 mW/m^2^) [43]. Interestingly, the AC composite with CB showed enhanced stability compared to AC and Pt as the air-cathode. After five months of operation, the MFCs operating with 10% CB/AC composite air-cathode produced the highest maximum power density of 1450 ± 10 mW/m^2^, which was 150% higher than that of the Pt (570 ± 30 mW/m^2^) and 14% higher than that of the plain activated cathodes (1270 ± 80 mW/m^2^) (Figure 1). The decrease in the catalytic activity of the Pt catalyst was associated mainly with biofouling and catalyst leaching, which can be avoided using carbon cloth (CC) as the catalyst support [43].

**Figure 2:**
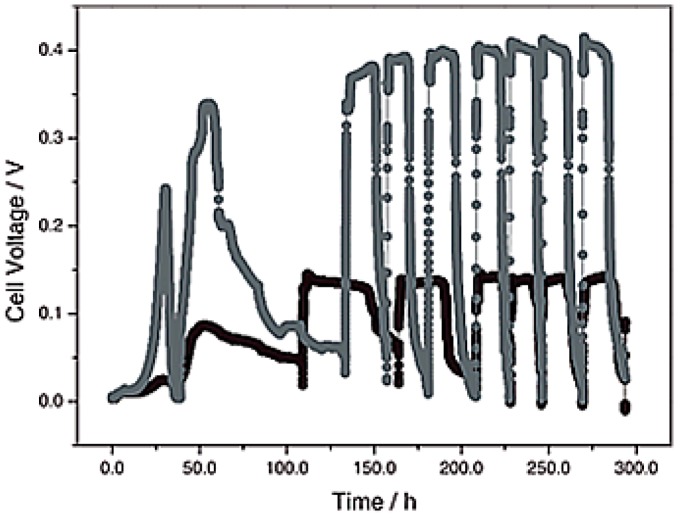
Voltage generation curve for MFCs operated with polyaniline (PANI) nanofibers (●) and PANI nanofibers/CB composite (●) cathodes. (Figure 4B from [72]. Reproduced by permission of The Electrochemical Society).

Carbon black served as a versatile support for various metal-based ORR catalysts, including noble metals, such as Pt [72]; organometalic complex, such as cobalt naphthalocyanine (CoNPc) [73] and FePc [37]; various metal oxides such as ZrO_2_ [74], Mn_2_O_3_, and Fe_2_O_3_ [68]; and conducting polymers [75]. Carbon black is also utilized as an inexpensive filler material in the polymer diffusion layer over a cathode catalyst to improve its oxygen permeability [76]. When CB is used as diffusion backing and a current collector, 3D stainless steel (SS) felt achieved 60% and 42% higher maximum power density compared to those using CC and SS mesh [77], respectively. Efforts have also been taken to fabricate an inexpensive cathode material using CB and polypyrrole (PPy) composite. Although the MFC operated with the PPy/CB composite as a cathode showed a lower power output than the Pt cathode, it is a more promising material based on the power per cost of the cathode material [15]. The incorporation of CB in the polymer matrix may play an important role in improving its physical and electrochemical properties [78]. Figure 2 represents the enhancement in the voltage output of MFCs using the PANI nanofibers/CB composite cathode compared to bare PANI nanofibers. The research on CB as a cathode material in MFCs has shown that the CB itself possesses very poor ORR activity but this can be enhanced drastically when combined with other ORR catalysts. Table 1 lists the performance of MFCs developed using CB as a component of the cathode materials.

### 7.2. Activated Carbon

Activated carbon is considered to be a more promising and inexpensive alternative cathode material compared to metal-based ORR catalysts. Although Pt-catalyst possesses the highest ORR overpotential reduction, AC has comparable or better performance in real MFC applications with the air-cathode system. In addition, the fabrication method of AC air-cathode largely affects its ORR property. (1) The brushing method, which is used widely and involves the application of a mixture of catalyst and binder on the surface of the support using a brush; (2) rolling method, which has attracted considerable attention recently; and (3) the dipping method, which has been newly introduced and used as a wet-cathode, are the available methods to fabricate AC cathodes [82]. The electron transfer numbers of ORR for different commercially available AC made from coal, peat, coconut shell, hardwood and phenolic resin varied from 2.1 (hardwood-based) to 3.6 (peat-based) and resulted in a power density of 630 to 1620 mW/m^2^ in MFCs [18]. In a similar report, the authors observed various contradictory observations compared to previous reports, such as the reverse trend for the relationship between the surface area of AC and the power density produced by MFCs using AC as the air-cathode. The AC produced from bituminous coal, which had the lowest surface area, produced the highest power density in the MFC tests, and the AC obtained from phenolic resin, which had the highest surface area, produced one of the lowest power densities. Interestingly, the actual performance of the AC cathode is independent of its electron transfer number and ORR potential measured using a RDE. One of the reasons for the above observation is that the diffusion limitation and concentration gradient exist in the real MFC applications and is absent in the RDE measurement. Dong et al. reported that the electron transfer number increased the values from 5% to 23% after the catalyst was prepared from AC powders using a rolling method with polytetrafluoroethylene (PTFE), while it decreased by 38% for the Pt/C powder electrode cast by the brushing method with Nafion [48]. Similarly, the AC cathode prepared on nickel foam using a PTFE binder produced a higher power density (1220 mW/m^2^) than the Pt-based cathode (1060 mW/m^2^) [83]. The abundant microporosity is also beneficial for the ORR activity providing more catalytic sites. The basic pretreatment using KOH to AC leads to up to 19% (804 to 957 mW/m^2^) improvement in the power density by increasing the surface conductivity, microporosity, and accelerating the charge transfer process. In contrast, a reverse trend was reported with an acidic pretreatment using HNO_3_, which causes a drastic decrease in the power density by 33% (804 to 537 mW/m^2^) [84]. The acid-pretreated AC also decreased the pH of the catholyte and formed corrosion on the interface of the AC catalyst and SS current collector [84]. Cheng et al. reported comparable power density using a MFC with nickel foam-coated AC to that produced by a Pt-CC cathode under the same conditions but the cost of a nickel foam AC cathode ($50/m^2^) was 1/30th that of a Pt-CC cathode ($1500/m^2^) [85].

**Figure 3:**
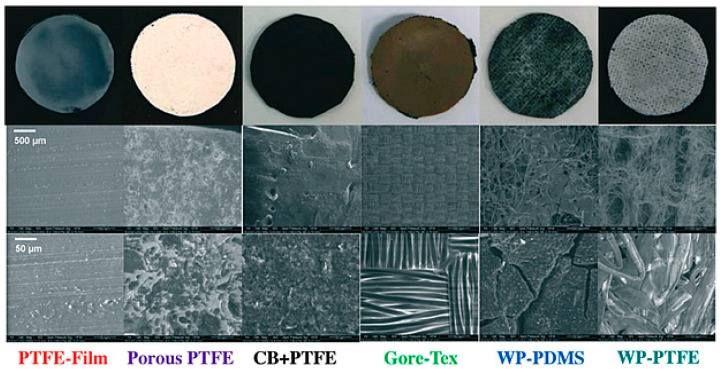
Digital photographs (**top row**) and scanning electron microscope (SEM) images (**bottom rows**) of different cathode diffusion layers. (Reproduced from [91] with permission of The Royal Society of Chemistry). PTFE: Polytetrafluoroethylene; WP: Wipe-based; PDMS: Polydimethylsiloxane.

**Figure 4:**
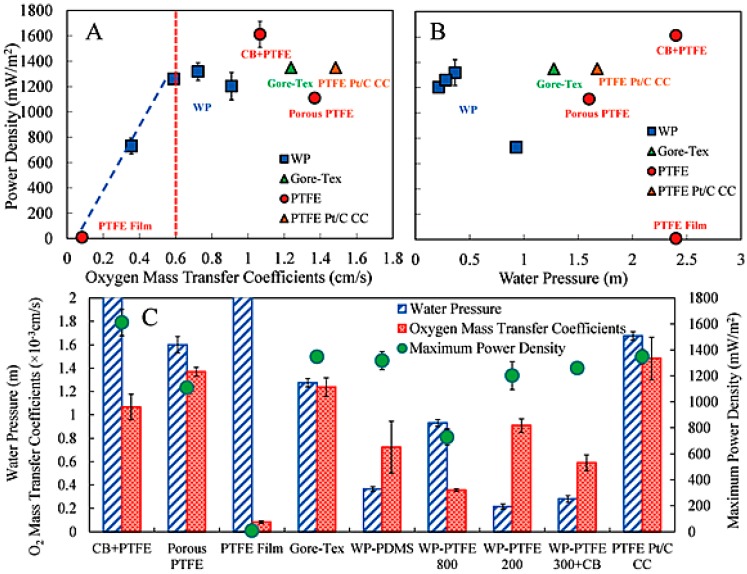
Maximum power density, oxygen diffusion coefficient, and water pressure of cathodes with different diffusion layers with the corresponding other characteristics. (**A**) Maximum power density vs. oxygen diffusion coefficient; (**B**) Maximum power density vs. water pressure; (**C**) Maximum power density, oxygen diffusion coefficient, and water pressure of cathodes with different diffusion layers. (Reproduced from [91] with permission of The Royal Society of Chemistry).

Zhang et al. tested an air-cathode [83], where PTFE-bonded AC was pressed as a catalyst layer, and reported excellent performance in a single chambered MFC compared to the Pt catalyst. Attempts have also been made to improve the performance of the AC air-cathode by surface modifications using quaternary ammonium salts which accelerate the OH^−^ transport [86]. Modification of the AC cathode with quaternary ammonium salts was also found be beneficial by limiting the growth of a cathodic biofilm, which is the major limitation of the membrane-free MFC technique [87]. The practical applications of ceramic-based MFCs using AC as the internal cathode were demonstrated by charging a mobile phone and running a direct current (DC) motor [88]. PTFE is used extensively as an inexpensive binder to fabricate the air-cathode with an AC layer on various supports including CC, SS, nickel foam, carbon fibers, etc. [89]. In the case of the large scale application of an AC air-cathode, the waterproofing of MFCs without affecting the oxygen permeability is an important task. The diffusion layer is coated on the opposite site of the AC catalyst and faces towards the air side, acting as a barrier for water leakage. Numerous choices of polymers are available to cast the diffusion layer, including PTFE, poly(vinylidene fluoride) (PVDF), poly(vinylidene fluoride-*co*-hexafluoropropylene) (PVDF-HFP), and poly(dimethylsiloxane) (PDMS). A carbon-free PVDF membrane synthesized using a simple phase inversion process provides good stability, 1.9 m (19 kPa), without a mesh supporter [90]. Zhang et al. fabricated AC cathodes coated with diffusion layers prepared from different polymer compositions (Figure 3) and tested them for MFC applications [91]. Among the all diffusion layers tested, the conductive CB/PTFE diffusion layer showed the most promising results, as the cathode fabricated with this layer had both the highest maximum power density and the highest water tolerance with a moderate oxygen mass transfer coefficient (Figure 4). Table 2 lists the performance of MFCs developed using the AC cathode.

### 7.3. Carbon Nanomaterials

Recently, carbon nanomaterials were introduced in most research fields owing to their remarkable properties that have also contributed in the field of MFCs as part of the anode, cathode and membrane materials particularly due to its high electrical conductivity and easily modified surface properties [98]. Instead of the direct application of carbon nanomaterials as a single component, its use as a modifier or composite with another catalyst is a more practical approach because of its high cost [99].

#### 7.3.1. Graphene

Graphene, a two-dimensional honeycomb-like network of sp^2^ hybridized carbon atom [100,101], has attracted considerable concern in the field of fundamental science and applied research [102]. A simple coating of graphene using a Nafion binder on a CC cathode enhanced ORR activity significantly compared to the bare CC but the activity was lower than that of the Pt/CB-modified cathode [55]. The graphene support also offers stability with enhancement in the ORR activity to the Pt catalyst [103]. Graphene works as an ORR catalyst in a composite with PANI and the appropriate addition of graphene improved the power density 116 times higher than that of the graphite felt (GF) cathode by reducing the charge transfer and ohmic resistance [104]. Compared to the other carbon materials, graphene is a better support for metal-based ORR catalysts. Table 3 lists the performance of MFCs operated with MnO_2_ supported on different carbon materials as the air-cathode. Graphene-supported MnO_2_ nanotubes cathodes performed 17% poorer than the Pt/C catalyst but still produced a higher performance than that supported with other carbon materials owing to the higher conductivity [105].

#### 7.3.2. Carbon Nanotubes

A previous study reported that the use of CNTs in a composite with Pt as the cathode helps reduce the use of Pt by 25% by boosting the performance of MFCs from 9% to 32% [106]. The electrochemical study showed that the improvement in the ORR activity is the key reason behind the enhancement of the MFC performance. The utilization of a CNT mat as a 3D air-cathode improved the power density two-fold, from 151 to 329 mW/m^2^, compared to CC, and could be increased further to 1118 mW/m^2^ by the addition of Pt [107]. The same study also described the impact of the preparation method of CNTs, which determined the quality or crystallization and electrical conductivity of CNTs on its performance as a catalyst support. Table 4 lists the results reported by Wang et al. for the different CNTs-based air-cathode-driven MFCs [108]. The 3D structure offered by the CNT mat provides a higher surface area for Pt accommodation inside the electrode space rather than on the surface, leading to increased reaction kinetics. The increased surface area through the CNT loading resulted in a higher limiting current density and better electrochemical performance. The ORR catalytic features of the biofilm-based cathode improved with the addition of CNTs through enhancement in the charge distribution and propagation within the biofilm [109].

**Figure 5:**
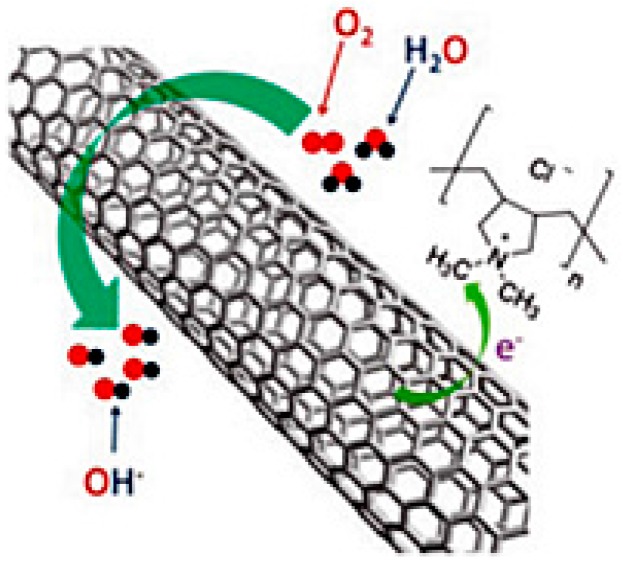
Illustration of the charge transfer process and ORR on poly(diallyl dimethyl ammonium chloride) (PDDA)-CNT. (Reprinted with permission from Ref. [115]. Copyright (2011) American Chemical Society).

The CNT composite with PPy is another good choice for comparatively low-cost cathode material for MFCs providing comparable output to the Pt catalyst [109]. The use of SS instead of CC or CP as a catalyst support and the current collector is a better alternative to reducing the principle cost of MFCs without altering the final performance. Zhang et al. reported a simple and scalable deep and dry method for the fabrication of a CNT-coated SS bio-cathode showing 49 times higher performance (147 mW/m^2^) than a bare SS cathode (3 mW/m^2^) [110]. Similarly, an improvement in the ORR activity was also noticed after the modification of GF using MWCNTs in the case of sediment MFCs [111]. In addition, most studies reported a lower ORR activity of the CNT-based cathode. Song et al. reported a new dual-layered approach by casting a hydrophilic layer by modified MWCNTs with Nafion to improve the ORR activity by significant reduction in the cathodic overpotential and performance by 140% compared to that of the MFC with commercial graphite cloth containing Pt [112]. Although the direct use of CNTs as an ORR catalyst is not impressive, its composite with different ORR catalysts is a good choice to enhance the ORR activity through improved charge transfer, electrochemical properties, and higher surface area [99,113,114]. Interestingly, Jiang at al. reported a PANI/MWCNTs composite as an alternative ORR catalyst with a maximum power density of 476 mW/m^2^, which was higher than the maximum power density of 367 mW/m^2^ obtained using pure MWCNTs but lower than that with a Pt/C cathode (541 mW/m^2^) [64]. Moreover, they also reported a very high power density, 465 mW/m^2^ using bare PANI as a cathode. This is a better approach for lowering the MFC cost as the cost of PANI and PANI/MWCNTs composite are considerably lower than the Pt/C catalyst. The experimental studies and quantum mechanics showed that the positively charged carbon atoms increased the ORR catalytic activity through charge-induced favorable O_2_ adsorption and the easy acceptance of electrons from the anode [56]. The physical adsorption of poly(diallyldimethylammonium chloride) (PDDA) on CNTs offers a strong electron-accepting ability to withdraw electrons from carbon atoms to induce the net positive charge and facilitate the ORR catalytic activity (Figure 5). The authors tested the durability using a chronoamperometric technique and found PDDA-CNT to be more consistent with its performance compared to Pt/C [115]. In addition, Wu et al. developed air-cathode using single-walled carbon nanotubes (SWCNTs) functionalized with polyelectrolytes (i.e., PDDA and poly[bis(2-chloroethyl)ether-alt-1,3-bis[3-(dimethylamino)propyl]urea] (PEPU) and tested for MFC applications) [116]. The PEPU-SWCNTs composite-based MFCs showed better performance with a maximum power density of 270.1 mW/m^2^, which was reasonably lower than that of the Pt/C catalyst (375.3 mW/m^2^), while the PDDA-SWCNTs composite-based MFCs produced 188.9 mW/m^2^. Table 5 lists the performance of MFCs developed using graphene and CNT-based cathode materials.

### 7.4. Heteroatom-Doped Carbon Materials

#### 7.4.1. N-Doped Carbon

**Figure 6:**
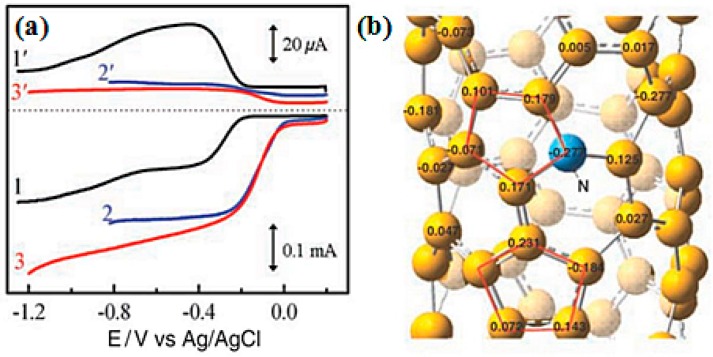
(**a**) Rotating ring-disk electrode (RRDE) voltammograms and the corresponding amperometric responses for oxygen reduction in air-saturated 0.1 M KOH at the CNT/GC (curves 1 and 1′), Pt-C/GC (curves 2 and 2′), and N-doped CNT/GC (curves 3 and 3′) electrodes at a scan rate of 10 mV/s. The electrode rotation rate was 1400 rpm, and the Pt ring electrode was poised at 0.5 V; (**b**) Calculated charge density distribution of the N-doped CNTs. (From [56]. Reprinted with permission from The American Association for the Advancement of Science).

The field of the N-doped carbon materials as an ORR catalyst was boosted drastically after an examination of the exceptional ORR catalytic activity of N-doped CNT by Gong et al. in 2009 [56]. Since then, various N-doped ORR catalysts have been developed and executed in fuel cell applications. This article is not the first evidence of the enhancement in the ORR activity by the introduction of N in the carbon architecture [125,126], but a more precise report about the activity. All available reports on N-doped carbon-based ORR catalysts are difficult to summarize. The breakthrough achievements in the N-doped carbon ORR catalysts and those applied in MFCs are discussed in this section. The N-doped carbon ORR catalyst can catalyze the direct reduction of oxygen to water via a four-electron pathway with much higher electrocatalytic activity, unlike undoped carbon materials, which generally follow a two-electron pathway with hydrogen peroxide formation. RDE analysis was carried out for N-doped CNTs and undoped CNTs (Figure 6a) showed that the transferred electron number per oxygen molecule involved in the ORR was 1.8 (at a potential of −0.40 V), and 3.9 (at a potential of −0.30 V), respectively. Quantum mechanics calculations revealed the mechanism behind the enhancement in the ORR catalytic activity after N-doping of the carbon texture. The strong electron affinity of the N atoms resulted in a significantly high positive charge density on the adjacent carbon atoms (Figure 6b). The change in the chemisorptions mode of O_2_ from the usual end-on adsorption (Pauling model) to a side-on adsorption (Yeager model) resulted in the parallel diatomic adsorption that effectively weakened O–O bonding to facilitate the ORR. Besides the high catalytic ORR activity, the N-doped carbon materials showed exceptional cycling durability and resistance to catalytic poisoning from methanol, CO, and sulfides [56].

**Figure 7:**
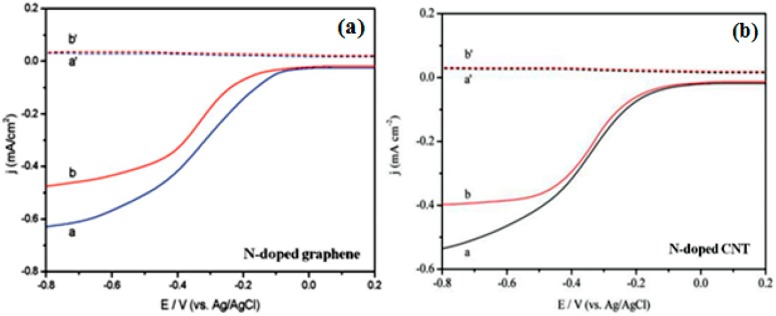
Rotating ring-disk electrode (RRDE) voltammograms of (**a**) N-doped graphene (curves a and a′) and Pt/C (curves b and b′) electrodes at a rotation rate of 1200 rpm (Reprinted with permission from [81]. Copyright (2011) American Chemical Society). (**b**) N-doped CNTs (a and a′, black line) and Pt/C (b and b′, red line) electrodes at a rotation rate of 1000 rpm. The electrolyte used for both RRDE voltammograms was an O_2_-saturated 50 mM PBS solution (pH 7.0). (Reproduced from [54] with permission of The Royal Society of Chemistry).

**Figure 8:**
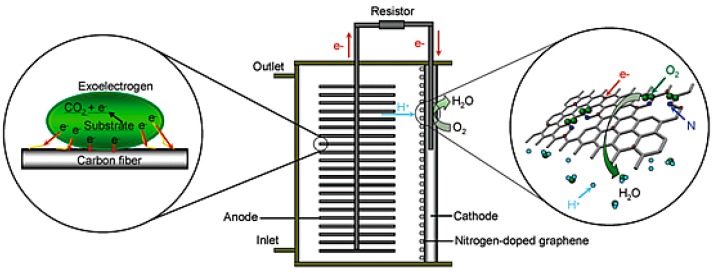
Schematic diagram of a membrane-free single chamber MFC operated with N-doped graphene coated carbon cloth (CC) as the air-cathode showing the basic mechanism of current generation. (Reprinted with permission from Ref. [81]. Copyright (2011) American Chemical Society).

One year after the ORR activity of N-doped CNTs was reported, Qu et al. examined the ORR activity of N-doped graphene synthesized using chemical vapor deposition (CVD) of methane in the presence of ammonia [9]. Similar to N-doped CNT, N-doped graphene showed a one step, four-electron pathway for the ORR activity with cycling durability and resistance to catalytic poisoning from methanol and CO. Feng et al. investigated N-doped graphene [81] and N-doped CNTs [54] as an ORR catalyst for the MFC in 2011. N-doped graphene synthesized using the detonation of a cyanuric chloride and trinitrophenol mixture and N-doped CNTs prepared by CVD have been demonstrated as a metal-free efficient ORR catalyst for power generation using MFCs. In both cases, the rotating ring-disk electrode (RRDE) analysis (Figure 7) confirmed the single step, four-electron transfer pathway for the ORR with 3.7 and 3.8 electrons transferred per oxygen molecule, respectively, which is similar to commercial Pt/C. The power density observed for the MFCs operated with the N-doped graphene air-cathode (1350 ± 15 mW/m^2^) was comparable to that of Pt/C (1420 ± 25 mW/m^2^), whereas the N-doped CNTs air-cathode showed a higher power output (1600 ± 50 mW/m^2^) than Pt/C (1393 ± 35 mW/m^2^) with better cyclic stability. Figure 8 presents the MFC operation and ORR mechanism using N-doped graphene-coated CC air-cathode.

The large scale synthesis of the catalyst is required as a concern with its practical applications. The gram-scale in situ synthesis of N-doped graphene was reported by Feng et al. and applied as ORR catalyst in MFC operated at neutral pH (Figure 8) [81]. Although the synthesis method required a comparatively lower temperature, the high pressure generated suddenly due to the detonation principle used in synthesis needs to be considered during synthesis. The MFC operated using the N-doped graphene air-cathode exhibited similar performance (power density of 1350 ± 15 mW/m^2^) with an 8% decrease after 35 cycles (~70 days) compared to the commercial Pt/C catalyst (1420 ± 25 mW/m^2^) with a 17% decrease after similar numbers of cycles. Most of the ORR catalysts are in powder form and require further treatment to coat on the desired support, which uses an expensive binder like Nafion. The synthesis of N-doped CNFs using pyridine with the CVD method is an alternative way to fabricate a binder-free N-doped carbon-based cathode for double chamber MFC [127]. Although the cathode obtained showed comparable performance to the Pt-decorated one, the open circuit potential of the N-doped cathode (0.5 V) was significantly lower than that of the Pt-decorated cathode (0.75 V).

**Figure 9:**
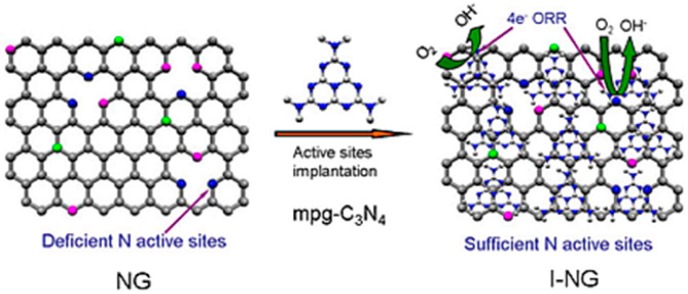
Schematic diagrams of the synergistic interactions between N-doped graphene and mesoporous carbon nitride (mpg-C_3_N_4_). (Adapted by permission from Macmillan Publishers Ltd.: [Scientific Reports] [128], copyright (2013)).

Continuous efforts have been made to further enhance the ORR catalytic activity and the stability of N-doped carbon. Enhancement of the surface area/porosity and additional impregnation of the active sites through N-doping are promising approaches to achieve the target. The increase in the number of catalytic sites, i.e., N-doping with synergistic coupling interactions between N-doped graphene and g-C_3_N_4_ (Figure 9) improved the ORR activity significantly with 100% of the four-electron pathway and high durability (almost no activity change after 100,000 potential cycling) than the pristine one [128]. The transferred electron number per oxygen molecule at −0.10 V was calculated to be 4.0 for the modified N-doped graphene, which was higher than that of pristine N-doped graphene (3.7) and Pt/C (3.9). Liu et al. suggested that the presence of more hydroxyl groups on N-doped carbon is responsible for their long term stability by repelling the proton [129]. As mentioned earlier, the presence of microporosity is advantageous for the ORR activity of AC. Recently, the preparation of N-doped carbon with microporous textural properties and a high surface area, 1398 m^2^/g, were synthesized using KOH-mediated chemical activation of graphene oxide and PANI composite [46]. The formation of porosity in the N-doped carbon matrix improved its ORR catalytic properties through the higher catalytic area/sites and produced a significantly higher power density (1159 mW/m^2^), i.e., 35% than that of commercial Pt/C. A similar strategy of chemical activation was utilized by Yang et al. to create the porosity in the N-doped CNFs and the 1.5 times higher performance of MFCs operated with porous N-doped CNFs was observed compared to that of the pristine N-doped carbon [130]. In contrast, the doping of N into the AC using N-containing precursor, such as cyanamide with simple sintering is an alternative and simple way to achieve the desired target [57]. Reflux with nitric acid [131], transfer-doping with PANI [132] and the sintering of metal-organic frameworks (MOFs) [133], are alternative methodologies reported for production of the N-doped carbon materials as the ORR catalyst for MFC applications.

#### 7.4.2. Other Heteroatom-Doped Carbon Materials

**Figure 10:**
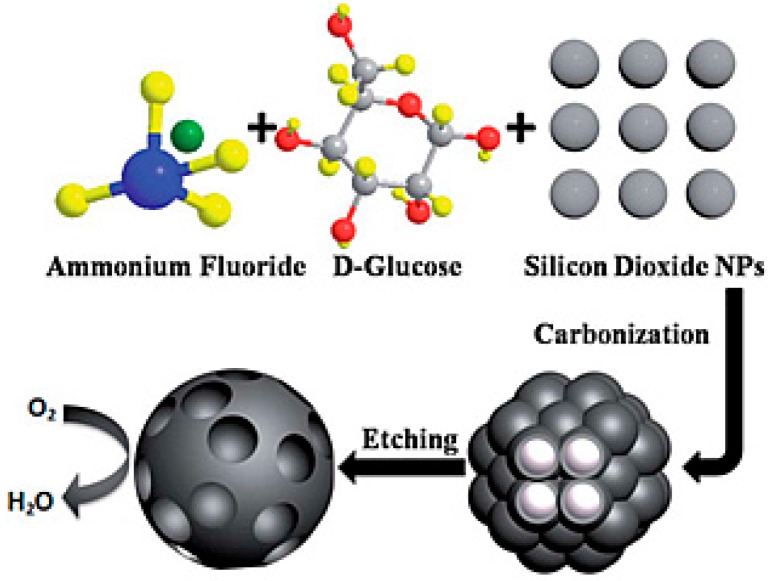
Schematic diagram for the synthesis of Si-F-doped porous carbon material. (Reproduced from [143] with permission of The Royal Society of Chemistry).

Simulation studies have confirmed that the formation of charge sites in the carbon architecture by disturbing the electro-neutrality of graphitic materials through the doping of a foreign atom is a key factor in enhancing the ORR activity, regardless of the dopants [134]. After observing the exceptional ORR activity of N-doped carbon materials, the influence of doping with different heteroatoms in the carbon texture on their ORR activity has been studied. The ORR activity of most heteroatom-doped carbon materials (except N) have been reported in alkaline medium but very few reports are available on their practical use in MFCs [135,136,137]. Orthophosphoric acid is commonly used as phosphorus precursor to achieve P-doping in the carbon texture [63]. Using this precursor, the P-doped carbon can be prepared either by in situ synthesis (mixed with carbon precursor followed by sintering) or by post-treatment modification. Liu et al. reported modified AC, which was prepared by a post-treatment with orthophosphoric acid as an efficient ORR catalyst for MFCs [138]. The presence of P played a key role in promoting the adsorption of oxygen and improving the ORR kinetics [139]. The MFC with a P-doped AC showed a maximum power density of 1278 ± 56 mW/m^2^, which was almost 1.3 times higher than that of the Pt/C cathode (980.5 ± 7 mW/m^2^) and the N-doped carbon cathode (934.7 ± 5 mW/m^2^). The decrease in the diffusion resistance due to the P-functional groups and lower charge transfer resistance by enhancing the diffusion of both H^+^ and oxygen promotes the ORR property of P-doped carbon. In the case of P-doping on AC, although the surface area decreased with increasing calcination temperature and micropores deform to mesopores, the ORR activity increased with increasing P-content. The enhancement in the ORR activity by the introduction of P-groups in AC is dominant on the negative impact of the decrease in the surface area [138]. Unlike N, P has a lower electron density than carbon and might act as a favorable adsorption site for oxygen with a partial positive charge. The sintering of cellulose with ammonium chloride and orthophosphoric acid resulted in a mixed N- and P-doped carbon, exhibiting higher catalytic activity than the individually doped carbon materials and Pt/C [140]. Similarly, Zhong et al. operated MFCs using N- and B-doped carbon nanoparticles as the air-cathode, which was synthesized using the coating-sintering route with dopamine and 3-aminophenyl boronic acid as the N- and B-precursors, respectively [141]. Owing to the highest electronegativity of fluorine, it has also been tested as a dopant to enhance the ORR activity of carbon materials and found to be more effective than Pt/C [142]. The synthesis of decorated porous carbon with Si-C-F decoration could be achieved by a simple silica nanoparticles template approach with glucose and ammonium fluoride (Figure 10). The charge distribution due to the synergistic effect of silicon and fluorine doping resulted in outstanding electrocatalytic performance and showed a maximum power density of 1026 mW/m^2^ [143].

Based on the charge density theory for the ORR activity, the dopant atom must possess a comparable difference in electronegativity to carbon (like N and P). In contrast, Yang et al. observed the ORR activity for S-doped graphene, which could not be explained based on charge density theory due to the similar electronegativity of C and S [137]. They used the spin density theory put forward by Zhang et al. to explain the ORR activity of S-doped graphene [144]. The porous carbon nanosheets decorated with N and S dopants showed better performance as an ORR catalyst in MFCs compared to individually (N- or S-) doped carbon nanosheets but 35% lower than that of Pt/C [66]. The AC fibers obtained from spider silk showed a uniform doping of N and S and an alternative way to obtain an efficient ORR catalyst from natural resources with a 1.56 times higher MFC output than that of the Pt/C cathode [145]. Table 6 lists the performance of the MFCs developed using different heteroatom-doped carbon materials as a cathode.

### 7.5. Carbon Materials from Sustainable Precursor

As discussed in previous sections, most carbon-based ORR catalysts are either nanomaterials, such as graphene and CNTs, or derived from commercial chemical reagents, which are expensive and require multi-step processing. Biomass contains a large amount of carbon and a simple calcination process can convert it to carbon materials. Hence, the utilization of biomass or carbon-based waste materials for the production of ORR catalysts would be a low cost and sustainable approach that has attracted the research community in recent years. Biomass, such as seaweed, microorganisms, plant mass, and bacterial cellulose, have been used to produce N-doped carbon-based ORR catalysts [59,60,61,62]. As this article is limited to MFC applications, this section discusses the ORR catalysts developed from biomass and used in MFCs.

Rice straw is an inexpensive and easily available biomass and used as a precursor for the preparation of N-doped carbon. After the carbonization of rice straw using a hydrothermal treatment followed by freeze drying, an ammonia treatment was performed at different temperatures to incorporate N-doping [150]. A hydrothermal treatment is necessary to reduce the oxygen and hydrogen content before the final calcination process to improve the carbon yield. In this study, the ORR activity of N-doped carbon was not correlated with the surface area and N content. N-doped carbon obtained at 900 °C with an intermediate N-content and surface area showed the best ORR catalytic property among the other catalysts prepared that was also higher than that of Pt/C with 3.86 electron transfer per oxygen molecule, suggesting the four-electron pathway during the ORR. Yuan et al. reported the synthesis of N-doped carbon using the chemical activation of biochar obtained from the banana plant [151]. Moreover, a hydrothermal treatment followed by annealing under N_2_ gas to plant moss (Weisiopsisanomala), a phylum of small and soft plants, commonly available plant species, could produce carbon nanoparticle-decorated N-doped porous carbon, which also served as a superior ORR catalyst in MFCs [152]. The organic molecules released from the moss create nanopores and are transformed into carbon nanodots during a hydrothermal treatment. The decoration of N-doped carbon with carbon nanoparticles helps increase the surface area and is also supportive to the ORR catalysis. Similar to rice straw-derived N-doped carbon, the catalyst prepared under intermediate preparation conditions (i.e., annealing at 900 °C showed better ORR activity and slightly higher MFC performance than Pt/C). The higher graphitic N content in the catalyst obtained at 900 °C, which further decreased with increasing annealing temperature and total N content, might explain the annealing temperature-dependent ORR catalytic activity of N-doped carbon [152].

## 8. Conclusions and Perspectives

The practical application of MFCs is limited mainly by its cost and performance. In both cases, the cathode plays a vital role. Cathodes consisting of Pt as the ORR catalyst performed well but they contribute to almost half of the total cost of MFCs. In this regard, carbon materials offer a cost effective approach by contributing significant ORR catalytic activity or supporting other ORR catalysts with a large surface area. This paper thoroughly reviewed the performance and viability of different carbon-based materials as efficient ORR catalysts for MFC applications. As a single chamber MFC is considered to be a more practical approach towards a large scale application, AC is a very good alternative for the Pt-based air-cathode with its remarkable ORR properties, which can be enhanced further by the addition of CB. Carbon nanotubes and graphene are a new class of carbon material with amazing electronic properties that offer enhancement in the ORR property when combined with other catalysts. Carbon materials are chemically more inert than metal-based ORR catalysts and provide long-term stability without significant loss of MFC performance. The rough surface of carbon materials exhibit various surface functionalities that can be modified easily by simple modification treatments. The type of surface groups on carbon materials can vary the ORR properties drastically and be controlled easily to achieve the best performance. The surface functionality of carbon materials not only contributes to the ORR properties, but also lowers the ohmic resistance by facilitating ion transfer. The formation of a positive charge in the carbon matrix through the doping of a heteroatom is a more constructive way to enhance the ORR performance of carbon materials with favorable O_2_ adsorption and weaker molecular bonding. Heteroatom doping with the simultaneous generation of porosity improved the ORR performance of the carbon catalyst due to the larger number of catalytic sites through the high surface area and porosity.

Although various carbon materials have been developed as ORR catalysts for MFCs with comparable catalytic activity, they requires a catalyst support, such as CP, cloth, and Ni-foam, which further contributes to the high cost of MFCs. Carbon-based materials with a self 3D rigid structure are a good alternative to overcome this problem. The fabrication and use of the free-standing 3D carbon materials for MFC applications have not received significant attention. Owing to the concerns with environmental issues and cost, the development of carbon-based ORR catalysts from biomass and waste materials will be an emerging trend in the MFC field. Despite the various reports on heteroatom-doped carbon ORR catalysts, the nature of the electro-catalytically active sites facilitating ORR and the origin of the catalytic reactivity are not completely understood. A precise study on the effects of heteroatom doping and textural properties, such as surface functional groups and porosity will be needed to better understand their role in ORR catalysis. The large scale production of carbon-based ORR catalysts with a low cost and environment-friendly manner is still a challenge and requires a more detail study. As the stability of the ORR catalyst is a very important parameter with the practical use of MFCs with lower maintenance cost, the long term applications of carbon-based catalysts in a real MFC study has received less attention. This review article shows that carbon-based materials are very promising ORR catalysts for the practical applications of MFCs and possess a broad scope in the area of wastewater treatment using MFCs and simultaneous power generation.

## Figures and Tables

**Table 1:** Summary of the performance of different MFCs run using cathode materials fabricated with CB in the composite with other oxygen reduction reaction (ORR) catalysts.

Catalysts	Catalyst Support	Casting Method	Power Output (mW/m^2^)	Performance Comparison	MFC Type	Bacteria Culture	Anode	Ref.
FePc/CB	CP	Drop casting	17.37	4.2 times vs. CP	Double chamber	*Enterobacter cloacae*	CP	[70]
FePc/CB (agitated)	CP	Drop casting	140.30	13.7 times vs. FePc	Double chamber	*Enterobacter cloacae*	CB	[70]
FePc/CB	CP	Drop casting	7.55	4.5 times vs. CP	Double chamber	Beer brewery wastewater	CP	[70]
FePc/CB (agitated)	CP	Drop casting	38.34	7.8 times vs. FePc	Double chamber	Beer brewery wastewater	CB	[70]
CoNPc/CB	CP	Brush casting	64.7	0.8 times vs. Pt/C 2.2 times vs. NPc/CB 6.9 times vs. CB	Double chamber	Anaerobic digester sludge	CP	[73]
ZrO_2_/CB	CC	Brush casting	596	1.4 times vs. CB 0.6 times vs. Pt/C	Single chamber	domestic wastewater	GBr	[74]
CuPc/CB	CP	Brush casting	118.2	3.1 times vs. CB 2.1 times vs. Pc/CB 1.2 times vs. Ni/CB 0.99 times vs. Pt/C	Double chamber	Palm oil effluent	CP	[79]
Nitric acid-treated CB	Membrane	Spray casting	170	3.3 times vs. CB 0.8 times vs. Pc/CB	Single chamber	Anaerobic sludge	CF	[80]
10% CB/AC	SS mesh	Spoon casting	1560	1.2 times vs. AC 1.1 times vs. 2% CB/AC 1.0 times vs. 5% CB/AC 1.0 times vs. 15% CB/AC 1.1 times vs. Pt/C	Single chamber	Pre-acclimated	GBr	[43]
PANI nanofiber/CB	CC	Brush casting	496	2.7 times vs. PANI nanofiber 0.8 times vs. Pt/C	Single chamber	Anaerobic sludge	CC	[81]
PPy/CB	CC	-	401.8	4.4 times vs. CB 0.7 times vs. Pt/C	Single chamber	Activated sludge	CC	[15]

CP: Carbon paper; CC: Carbon cloth; CB: Carbon black; GBr: Graphite brush; CF: Carbon felt; FePc: Iron phthalocyamine; PPy: Polypyrrole; CuPc: Copper phthalocyamine; CoNPc: Cobalt naphthalocyanine; NPc: Naphthalocyanine.

**Table 2:** Summary of the performance of different MFCs runs using AC as a cathode.

Catalyst	Catalyst Support	Casting Method	Power Output (mW/m^2^)	Performance Comparison	MFC Type	Bacteria Culture	Anode	Ref.
AC	SS mesh	Press	892	0.9 times vs. Pt/C (initial) 1.2 times vs. Pt/C (4 months)	Single chamber	Domestic wastewater	CBr	[92]
AC	SS mesh	Rollingpress	2348	-	Single chamber	Pre-acclimated	CBr	[93]
AC (heat-treated)	SS mesh	Press	1400	1.3 times vs. AC 1.1 times vs. AC/CB 1.1 times vs. Pt/C	Single chamber	Pre-acclimated	GBr	[67]
AC	SS mesh	Spatula	1430	1.3 times vs. Pt/C	Single chamber	Pre-acclimated	GBr	[76]
AC	SS mesh (40 mesh)	Rolling press	2151	1.5 times vs. AC on 80 Mesh SS	Single chamber	Domestic wastewater	CBr	[94]
Modified AC	SS mesh	Rolling	892	1.3 times vs. AC	Single chamber	Domestic wastewater	CBr	[95]
Granular AC	-	-	676	1.8 times vs. semi-coke 5.5 times vs. graphite 11.2 times vs. CF	Packed-bed	Pre-acclimated	GBr	[96]
AC (coal-based)	SS mesh	Press	1620	Similar with AC (peat) 2.6 times vs. AC (hardwood) 0.8 times vs. Pt/C	Single chamber	Pre-acclimated	CBr	[18]
AC (KOH-treated)	SS mesh	Rollingpress	957	1.2 times vs. AC 1.8 times vs. AC (HNO_3_^-^treated)	Single chamber	Pre-acclimated	AC on SS mesh	[84]
AC (not sintered catalyst layer)	SS mesh	Rolling	1086	1.3 times vs. AC (sintered)	Single chamber	Pre-acclimated	CM	[97]
AC	Nickel foam	-	1190	0.9 times vs. Pt/C on CC	Single chamber	Pre-acclimated	CBr	[85]

CBr: Carbon brush; CM: Carbon mesh.

**Table 3:** Effect of the different carbon supports on the MnO_2_ nanotubes (0.3 gm/cm^2^) based air-cathode in the MFC and a comparison with the benchmark Pt/C [105].

Cathodes	Max. OCP (mV)	Max. Vol. Power Density (W/m^3^)	Max. Columbic Efficiency (%)	COD Removal Efficiency (%)	Internal Resistance (Ω)
Catalyst-free	677	0.57	5.0	69.2	172
MnO_2_-NTs/Vulcan XC	754	2.2	8.4	78.7	108
MnO_2_-NTs/MWCNTs	793	3.94	11.0	82.9	97
MnO_2_-NTs/graphene	812	4.68	11.5	83.7	85
Pt/C	839	5.67	12.6	84.4	75

OCP: Open circuit potential; COD: Chemical oxygen demand; NTs: Nanotubes; MWCNTs: Multiple-walled carbon nanotubes.

**Table 4:** Performance of the MFCs operated with different CNTs-based air-cathodes [107].

Electrode	Pt (0.5 mg/cm^2^) Coating Method	Power Density (mW/m^2^)
CC	-	151
CC-Pt	10% Pt/CB mixture (brush)	1071
CNT Mat	-	329
CNT Mat-Pt	10% Pt/CB mixture (brush)	1118
Single-walled CNTs (SWCNTs)		117
SWCNTs-Pt	Laboratory-synthesized SWCNTs; H_2_PtCl_6_ (Microwave)	302
SWCNTs-Pt	Commercial SWCNTs; H_2_PtCl_6_ (Microwave)	522
MWCNTs-Pt	Commercial MWCNTs; H_2_PtCl_6_ (Microwave)	174

SWCNTs: single-walled carbon nanotubes.

**Table 5:** Summary of the performances of different MFCs operated using graphene and CNT-based cathode materials.

Catalyst	Catalyst Support	Casting Method	Power Output (mW/m^2^)	Performance Comparison	MFC Type	Bacteria Culture	Anode	Ref.
r-Graphene oxide sheet	CC	-	2.9 (W/m^3^)	0.6 times vs. Pt/C	Double chamber	Anaerobic sludge	CBr	[55]
r-Graphene oxide sheet	CC	-	2.5 (W/m^3^)	8.3 times vs. CC	Double chamber	Anaerobic sludge	CBr	[55]
r-Graphene oxide particles	CC	-	3.3 (W/m^3^)	11.0 times vs. CC	Double chamber	Anaerobic sludge	CBr	[55]
Graphene/PANI	GF	In situ deposition	99	116.5 times vs. GF	Sediment	Residual sludge	Graphite	[104]
Graphene/MnO_2_	CP	Spray casting	4.68 (W/m^3^)	1.2 times vs. MWCNTs/MnO_2_ 2.1 times vs. CB/MnO_2_ 0.8 times vs. Pt/C	Single chamber	Anaerobic consortia	CC	[105]
Pt(15%)-Co/graphene	CC	-	1378	Almost similar with Pt/C (20%)	Single chamber	Domestic	CC	[13]
r-Graphene oxide /PEDOT/Fe_3_O_4_	CC	Spray casting	3525	8.2 times vs. CC 4.5 times vs. Fe_3_O_4_ 2.2 times vs. r-Graphene oxide/Fe_3_O_4_ 1.5 times vs. r-Graphene oxide/PEDOT	Single chamber	Anaerobic sludge	CC	[117]
r-Graphene oxide/SnO_2_	SS grid/CF	-	80	1.6 times vs. Pt/C	Single chamber	Seawater inoculums	CF	[118]
Graphene/MnO_2_	SS net	-	2084	6.2 times vs. non-catalyzed 1.4 times vs. MnO_2_ 1.2 times vs. Pt/C	Single chamber	Anaerobic sludge	CF	[119]
CNTs mat	-	-	329	2.2 times vs. CC	Single chamber	Exoelectrogenic bacteria	GBr	[107]
β-MnO_2_/CNT	CC	Spray casting	97.8	4.4 times vs. α-MnO_2_/CNT 1.2 times vs. γ-MnO_2_/CNT 0.6 times vs. Pt/C	Single chamber	Domestic wastewater	CC	[120]
Pt-Ni-MWCNTs	CC	-	1220	0.9 times vs. Pt/C	Single chamber	Pre­domesticated	CC	[121]
Pt/modified CNTs	Titanium mesh	Brush casting	911.3	2.0 times vs. Pt/C	Single chamber	Local pond	CF	[122]
CuSe/CNTs	CC	Brush casting	425.9	1.7 times vs. CNTs 1.6 times vs. CuSe 0.9 times vs. Pt/C	Single chamber	Activated sludge	CC	[114]
Dual layered CNTs	SS	Spray casting	207	2.4 times vs. Single layer CNTs 1.4 times vs. Pt/Graphite cloth	Single chamber	Anaerobic sludge	Graphite fabric	[112]
MWCNTs	GF	Electrophoretic deposition	214.7	1.6 times vs. GF	Sediment	-	GF	[110]
MWCNTs	SS net	Electrophoretic deposition	31.6	3.2 times vs. SS net	Sediment	-	SS net	[123]
MnO_2_/CNT	CP	In situ synthesis	210	2.3 times vs. MnO_2_/CNT (mechanical mixing) 0.9 times vs. Pt/C	Single chamber	Anaerobic sludge	GF	[124]
PEPU-SWCNTs	CC	Spray casting	270.1	2.3 times vs. PDDA-CNT 0.9 times vs. Pt/C	Single chamber	Activated sludge	CC	[117]

r-Graphene oxide: reduced graphene oxide.

**Table 6:** Summary of the performance of different MFCs operated using different heteroatom-doped carbon materials as a cathode.

Catalyst	Doping Agent/Method	Catalyst Support	Power Output (mW/m^2^)	Performance Comparison	MFC Type	Bacteria Culture	Anode	Ref.
N-CNFs (activated)	PPy	CP	1377	1.5 times vs. N-CNFs 4.0 times vs. CP	Double chamber	Sewage wastewater	CG	[130]
N-carbon powder (pre-treated)	HNO_3_ treatment	CC	934.7	Almost similar with Pt/C	Single chamber	Domestic sewage	CBr	[131]
N-doped graphene	Cyanuric chloride	CC	1350	Almost similar to Pt/C	Single chamber	Suspended bacteria	CBr	[81]
N-carbon powder	HNO_3_ treatment	CC	222.5	0.9 times vs. Pt/C 1.1 times vs. Pt/C (with Na_2_S)	Double chamber	Domestic wastewater	CC	[146]
N-graphene/C_3_N_4_	NH_4_OH, cyanamide	CC	1618	1.2 times vs. N-graphene 1.1 times vs. Pt/C	Single chamber	Suspended bacteria	CBr	[128]
Co_3_O_4_/N-graphene	NH_4_OH	ITO substrate	1340	0.9 times vs. Pt/C	Double chamber	*Shewanella oneidensis*, MR-1	CG	[147]
Fe-N-AC	Ethylenediamine	SS mesh	2437	2.1 times vs. AC	Single chamber	Domestic wastewater	CF	[148]
Mesoporous N-carbon	Ethylenediamine	CC	979	0.8 times vs. Pt/C	Single chamber	Domestic wastewater	GBr	[149]
N-CNT	Ethylenediamine	CC	1600	1.1 times vs. Pt/C	Single chamber	Domestic wastewater	CBr	[54]
N-carbon	NH_3_ gas	SS mesh	1041	2.5 times vs. undoped carbon 1.8 times vs. Pt/C	Single chamber	Domestic wastewater	GBr	[28]
Porous N-carbon nanosheets/graphene	PANI	SS net	1159	1.8 times vs. N-carbon 1.3 times vs. Pt/C 18.1 times vs. without catalyst	Single chamber	Anaerobic sludge	CBr	[46]
N-graphene	NH_4_OH	CP	776	Slightly higher than Pt/C	Double chamber	Activated sludge	CC	[129]
Acid/base treated N-AC	Cyanamide	CC	650	2.1 times vs. AC 1.6 times vs. N-AC 1.4 times vs. Pt/C 1.1 times vs. acid-treated N-AC	Double chamber	Digester effluent	CBr	[58]
N-/P-doped carbon	Ammonium phosphate	SS mesh	2293	2.9 times vs. N-carbon 2.6 times vs. P-carbon 1.4 times vs. Pt/C	Single chamber	Domestic wastewater	GBr	[63]
N-/F-CB	NH_3_, PTFE	SS mesh	672	1.4 times vs. undoped carbon 1.1 times vs. F-carbon 1.3 times vs. N-carbon 1.2 times vs. Pt/C	Single chamber	---	CM	[142]
P-AC	H_3_PO_4_	SS mesh	1278	1.75 times vs. untreated AC 1.5 times vs. AC heated at 800 °C	Single chamber	Domestic wastewater	CF	[138]
N-/S-CNF	Spider silk	SS mesh	1800	1.6 time vs. Pt/C	Single chamber	Domestic wastewater	GBr	[145]
N-/B-carbon nanoparticles	Polydopamine, Aminobenzene boronic acid	CC	642	0.9 time vs. Pt/C 1.2 times vs. N-carbon nanoparticles 5.3 times vs. carbon nanoparticles	Single chamber	Anaerobic sludge	CC	[142]
P-AC	H_3_PO_4_	SS mesh	1096	1.5 times vs. AC	Single chamber	Domestic wastewater	CF	[139]
P-carbon	Cellulose phosphate	SS mesh	1312	2.6 times vs. undoped carbon 1.1 times vs. Pt/C	Single chamber	Domestic wastewater	GBr	[140]
N-/S-carbon nanosheets	NH_3_, Diphenyl disulfide	CC	1500	0.65 times vs. Pt/C	Double chamber	-	CBr	[66]
Si-/F-porous carbon	SiO_2_, Ammonium fluoride	SS mesh	1026	1.1 times vs. Pt/C	Single chamber	Domestic wastewater	CM	[143]

CG: Carbon granule; ITO: Indium tin oxide.

## Abbreviations

AC	Activated carbon
CB	Carbon black
CBr	Carbon brush
CC	Carbon cloth
CF	Carbon felt
CG	Carbon granule
CM	Carbon mesh
CNFs	Carbon nanofibers
CoNPc	Cobalt naphthalocyanine
CNTs	Carbon nanotubes
COD	Chemical oxygen demand
CP	Carbon paper
CuPc	Copperphthalocyanine
CVD	Chemical vapor deposition
DC	Direct current
FePc	Iron phthalocyanine
GBr	Graphite brush
GC	Glassy carbon
ITO	Indium tin oxide
MFC	Microbial fuel cell
mpg-C_3_N_4_	Mesoporous carbon nitride
MOFs	Metal-organic frameworks
MWCNTs	Multi-walled carbon nanotubes
OCP	Open circuit potential
ORR	Oxygen reduction reaction
PANI	Polyaniline
PDDA	Polydiallyldimethylammonium chloride
PDMS	Poly(dimethylsiloxane)
PEDOT	Poly (3,4-ethylenedioxythiophene)
PEM	Proton-exchange membrane
PEPU	Poly[bis(2-chloroethyl)ether-alt-1,3-bis[3-(dimethylamino)propyl]urea]
PPy	Polypyrrole
PTFE	Polytetrafluoroethylene
PVDF	Poly(vinylidene fluoride)
PVDF-HFP	Poly(vinylidene fluoride-co-hexafluoropropylene)
RDE	Rotating disk electrode
r-Graphene oxide	Reduced graphene oxide
RRDE	Rotating ring-disk electrode
SEM	Scanning electron microscope
SS	Stainless steel
SWCNTs	Single-walled carbon nanotubes
WP	Wipe-based
